# Enhancing the cooling potential of photoluminescent materials through evaluation of thermal and transmission loss mechanisms

**DOI:** 10.1038/s41598-021-94354-7

**Published:** 2021-07-19

**Authors:** Samira Garshasbi, Shujuan Huang, Jan Valenta, Mat Santamouris

**Affiliations:** 1 Faculty of Built Environment, University of New South Wales, Sydney, 2052 Australia; 2 School of Engineering, Sustainable Energy Research Centre, Macquarie University, Sydney, NSW 2109 Australia; 3 Department of Chemical Physics and Optics, Faculty of Mathematics and Physics, Charles University, Prague, Czechia

**Keywords:** Optics and photonics, Physics

## Abstract

Photoluminescent materials are advanced cutting-edge heat-rejecting materials capable of reemitting a part of the absorbed light through radiative/non-thermal recombination of excited electrons to their ground energy state. Photoluminescent materials have recently been developed and tested as advanced non-white heat-rejecting materials for urban heat mitigation application. Photoluminescent materials has shown promising cooling potential for urban heat mitigation application, but further developments should be made to achieve optimal photoluminescence cooling potential. In this paper, an advanced mathematical model is developed to explore the most efficient methods to enhance the photoluminescence cooling potential through estimation of contribution of non-radiative mechanisms. The non-radiative recombination mechanisms include: (1) Transmission loss and (2) Thermal losses including thermalization, quenching, and Stokes shift. The results on transmission and thermal loss mechanisms could be used for systems solely relying on photoluminescence cooling, while the thermal loss estimations can be helpful to minimize the non-radiative losses of both integrated photoluminescent-near infrared (NIR) reflective and stand-alone photoluminescent systems. As per our results, the transmission loss is higher than thermal loss in photoluminescent materials with an absorption edge wavelength (*λ*_*AE*_) shorter than 794 nm and quantum yield (*QY*) of 50%. Our predictions show that thermalization loss overtakes quenching in photoluminescent materials with λ_AE_ longer than 834 nm and *QY* of 50%. The results also show that thermalization, quenching, and Stokes shift constitute around 56.8%, 35%, and 8.2% of the overall thermal loss. Results of this research can be used as a guide for the future research to enhance the photoluminescence cooling potential for urban heat mitigation application.

## Introduction

Heat rejecting coating materials as one of the most effective measures for urban overheating mitigation have been widely investigated during the last decades^1–4^. The light-coloured heat rejecting materials present high cooling potential but could not be used at a large scale due the aesthetic and glare considerations. The main strategy to enable the wide application of heat rejecting coatings for urban heat mitigation is to develop advanced coloured/non-white cool coatings to reject the incoming light with minimal thermal loss. Near-infrared (NIR)-reflective coatings as the first generation of non-white coatings are characterized by high solar reflection in NIR range. The NIR-reflective materials have the same colour as conventional construction materials due to their similar reflection in the visible range. Photoluminescent materials as the more recent heat rejecting coating technology are advanced materials capable of reemitting a portion of the absorbed energy through so-called photoluminescence (PL) effect^5–7^. PL effect refers to non-thermal/radiative relaxation of a portion of absorbed energy. Photoluminescent materials can be used to reject the incoming solar radiation at shorter wavelengths (e.g. UV and visible range). Photoluminescent materials can be classified as mineral photoluminescent materials and nano-scale photoluminescent materials (quantum dots (QDs)). QDs are nano-scale light-emitting semiconductor materials with intriguing adjustable photoluminescent properties due to the quantum confinement effect. PL effect occurs at wavelengths equal or shorter than absorption edge wavelength (*λ*_*AE*_) of the photoluminescent material^7,8^. The remaining part of the absorbed energy with wavelengths equal or shorter than λ_AE_ is released through thermal radiation mechanisms including thermalization, quenching, and Stokes shift^9–11^. The incident radiation with wavelengths longer than *λ*_*AE*_ is also transmitted through the photoluminescent layer, as it does not have the required energy to induce electronic excitation to the next energy level^9,12^. Assuming the substrate material to be a highly absorptive construction material, the transmitted light through photoluminescent layer is eventually be converted into heat as well. Most of the previous research on PL cooling are studies focusing on the experimental thermal performance evaluation of photoluminescent materials and their integration with NIR-reflective coatings for the urban overheating mitigation^5,13,14^. In one of our recent research papers, we have developed a mathematical model for precise estimation of PL cooling potential^7^. The proposed PL cooling model is a cost and time-effective method to distinguish the heat rejection through PL effect from that of reflection. Surface temperature reduction potential (i.e. surface temperature difference between the photoluminescent material and its corresponding non-photoluminescent counterpart) and reemitted energy (*Q*_*PL*_) were employed as photoluminescent cooling indicators in the study.

In this paper, we aim to gain a better insight on the radiative cooling mechanism of photoluminescent materials through computation of all involved non-radiative processes including thermal and transmission losses. To achieve this, we have extended our recently developed PL cooling model to study the impact of photoluminescent properties on thermal and transmission loss mechanisms^7^. Then, we have evaluated the contribution of thermal and transmission loss mechanisms compared to the overall non-radiative loss. At last, the most efficient methods for improving the PL cooling potential are proposed. Results of this research can determine the direction of future research on improving the PL cooling efficiency of photoluminescent materials.

## Photoluminescence cooling: thermal and transmission loss

Photoluminescence (PL) cooling refers to the non-thermal/radiative relaxation of absorbed energy of the surface coating. Fluorescence and phosphorescence are two forms of PL effect. In contrast to fluorescent materials that glow/reemit light under a continuous excitation light source, the phosphorescent materials can have afterglow effect that persists after the excitation light has been switched off. PL cooling occurs for the incident light with an energy level equal or higher than the bandgap energy. Photons with energies smaller than the bandgap are transmitted through the photoluminescent material^14,15^. In this paper, transmission loss refers to the total energy of incoming photons with energies smaller than the bandgap.

**Figure 1:**
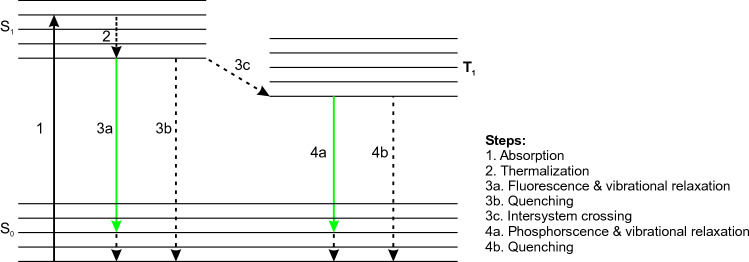
Schematic illustration of radiative and non-radiative/thermal loss mechanisms including thermalization, quenching, and Stokes shift for fluorescent and phosphorescent materials.

In parallel, photons with energies larger than bandgap are absorbed, but only a portion of their absorbed energy is reemitted via PL effect, and the remaining part is lost to heat (i.e. thermal loss). There are three major thermal loss mechanisms: 1. Thermalization, 2. Quenching, 3. Stokes shift. Thermalization loss, as one of the important thermal loss mechanisms, refers to the non-radiative/thermal relaxation of excess energy from excited energy band to the bottom of conduction band (See Fig. 1)^9,16^. The deexcitation process from conduction band to valence band is different for fluorescent and phosphorescent materials. As for the fluorescent materials, two scenarios may occur for electrons at conduction energy level: 1. In the first scenario, electrons recombine back to the singlet ground energy state through radiative process, while releasing the remaining energy in the form of vibrational relaxations. The radiative and vibrational relaxations are known as fluorescent cooling and Stokes shift loss, respectively; 2. The second scenario is the non-radiative deexcitation of electrons from conduction band to the ground energy state due to the quenching loss. Quenching loss refers to non-radiative relaxation of electrons to ground energy band caused by unwanted optical effects including defects and reabsorption^10,17^. Quantum yield (*QY*) is a key PL variable through which the number of reemitted photons can be determined. With regard to phosphorescent materials, electrons first transition from singlet excited state to triplet state through a non-radiative mechanism known as intersystem crossing. This is followed by radiative and/or non-radiative recombination of electrons to the ground energy state. The singlet to triplet state transition occurs due to the inversion of electrons spin. The radiative loss from triplet to singlet energy state is known as phosphorescent cooling. Since triplet to singlet is a spin forbidden transition in phosphorescent materials, it has a longer lifetime than the deexcitation process in fluorescent materials. Similar to the fluorescent materials, the electrons may deexcite non-radiatively due to quenching. *QY* efficiency of the phosphorescent material can be used to estimate the non-radiative loss caused by quenching. The Stokes shift loss in phosphorescent materials includes intersystem crossing and vibrational relaxations. The Stokes shift thermal loss as a commonly observed effect in fluorescent/phosphorescent materials occurs due to an optically passive state in valence band or formation of triplet state in conduction band^18^.

In addition to the thermal loss mechanisms, estimation of the transmission loss intensity is also of very high importance to explore the most efficient methods for enhancing the photoluminescence cooling potential. Transmission loss can be drastically reduced through up conversion of two low energy photons into one higher energy photon^12,19,20^. Likewise, thermalization loss could be decreased by using nanoparticle structures capable of converting one high energy photon into two low energy photons due to their down conversion properties^21,22^. Thermalization loss can also be minimalised by a tandem structure, i.e., stacking a larger bandgap material on top of smaller bandgap material^23,24^. As for photoluminescence materials with significant quenching loss, there are two methods to minimize the following thermal mechanisms: 1. Avoid reabsorption by controlling the distance between photoluminescence particles by embedding in a matrix host or surface ligands, 2. Control the surface-defect related thermal radiations in nano-scale photoluminescent materials (QDs) using core–shell QDs structures and surface ligand engineering of QDs^25–27^.

## Model description

In this paper, we have proposed an extended version of our predictive photoluminescence cooling model for computation of the non-radiative components including thermal and transmission loss mechanisms. The photoluminescence cooling model were developed according to the radiative mechanism in photoluminescence materials. Re-emitted energy and temperature difference between the photoluminescence and corresponding non- photoluminescence sample were the two main indicators for the photoluminescence cooling potential estimation^7^. Herein, the non-radiative processes are computed based on the equations on the reemitted energy index. The evaluation of thermal and transmission loss mechanisms in photoluminescence materials can give a general understanding of their contribution and potential methods to maximize the photoluminescence cooling potential. According to the photoluminescence cooling model, the non-thermal radiative recombination through PL effect can be written as follow^7^:

1 $$ E_{PL} = NP_{reemitted} \times E_{p} \left( {\lambda_{PL} } \right) $$

where *E*_*PL*_ is the reemitted energy by PL effect, *NP*_*reemitted*_ is the number of reemitted photons, and *E*_*p*_(*λ*_*PL*_) is the energy of each photon with PL peak wavelength (*λ*_*PL*_). *NP*_*reemitted*_ can be calculated by multiplying *QY* by the number of absorbed photons in the PL effect wavelength range between 300 nm and *λ*_*AE*_:

2 $$ NP_{reemitted} = QY \times \mathop \sum \limits_{\lambda = 300}^{{\lambda_{AE} }} NP_{absorbed} \left( \lambda \right) $$

where *QY* is quantum yield, and *NP*_*absorbed*_(*λ*) is the number of absorbed photons with a wavelength of *λ*. The number of absorbed photons can be obtained by the following equation:

3 $$ NP_{absorbed} \left( \lambda \right) = \frac{{E_{absorption} \left( \lambda \right)}}{{E_{p} \left( \lambda \right)}} $$

where *E*_*absorption*_(*λ*) is the total absorbed energy and *E*_*p*_(*λ*) is the energy of each photon with a wavelength of *λ*. *E*_*absorption*_(*λ*) and *E*_*p*_(*λ*) can be calculated as follow:

4 $$ E_{absorption} \left( \uplambda \right) = \alpha \cdot A \cdot I_{SW} \cdot \varphi \left( \uplambda \right) $$

5 $$ E_{P} \left( \uplambda \right) = \frac{{h_{Planck} \cdot C}}{\uplambda } $$

where *α* is solar absorption, *A* is surface area, *I*_*SW*_ is the shortwave solar irradiation (0.285–3 μm), and *φ*(*λ*) is the spectral distribution of global solar radiation according to global standard spectrum (AM1.5g). Also, *h*_*planck*_ is Planck constant (6.62 × 10^−34^ J s) and *C* is speed of light (3 × 10^8^ m s^−1^). By combining Eqs. (1–5), the overall equation for reemitted energy (*E*_*PL*_) can be written as follow:

6 $$ E_{PL} = \frac{QY}{{SS + \lambda_{AE} }} \cdot \left( {\mathop \sum \limits_{{{\uplambda } = 300}}^{{{\uplambda }_{AE} }} \uplambda \cdot \varphi \left( \uplambda \right)} \right) \cdot \alpha \cdot A \cdot I_{SW} $$

where SS (Stokes shift) can be calculated using the following equation:

7 $$ SS = \uplambda_{PL} - \uplambda_{AE} $$

The photoluminescence cooling occurs in wavelengths equal or shorter than *λ*_*AE*_. Therefore, the transmission loss can be computed by multiplying *NP*_*absorbed*_ by *E*_*p*_(*λ*) in the wavelength range between *λ*_*AE*+1_ and 2500:

8 $$ E_{transmission} = \mathop \sum \limits_{{\lambda = \lambda_{AE} + 1}}^{2500} NP_{absorbed} \left( \lambda \right) \times E_{p} \left( \lambda \right) = \left( {\mathop \sum \limits_{{\lambda = \lambda_{AE} + 1}}^{2500} \varphi \left( \lambda \right)} \right) \cdot \alpha \cdot A \cdot I_{SW} $$

As for computation of the thermal loss mechanisms, we first need to compute the total absorbed energy in photoluminescence cooling wavelength range (i.e. 300 nm to *λ*_*AE*_) using the following equation:

9 $$ E_{absorption} = \mathop \sum \limits_{\lambda = 300}^{{\lambda_{AE} }} NP_{absorbed} \left( \lambda \right) \times E_{p} \left( \lambda \right) = \left( {\mathop \sum \limits_{\uplambda = 300}^{{\uplambda_{AE} }} \varphi \left( \uplambda \right)} \right) \cdot \alpha \cdot A \cdot I_{SW} $$

The absorbed energy is composed of the bandgap and thermalization components. The bandgap and thermalization energies can be computed as follow:

10 $$ E_{absorption} = E_{g} + E_{thermalisation} $$

11 $$ E_{g} = E_{p} \left( {\lambda_{AE} } \right) \times \mathop \sum \limits_{\lambda = 300}^{{\lambda_{AE} }} NP_{absorbed} \left( \lambda \right) = \left( {\mathop \sum \limits_{{{\uplambda } = 300}}^{{{\uplambda }_{AE} }} \frac{{\uplambda }}{{\lambda_{AE} }} \cdot \varphi \left( {\uplambda } \right)} \right) \cdot \alpha \cdot A \cdot I_{SW} $$

12 $$ E_{thermalisation} = \mathop \sum \limits_{\lambda = 300}^{{\lambda_{AE} }} NP_{absorbed} \left( \lambda \right)\left( {E_{p} \left( \lambda \right) - E_{p} \left( {\lambda_{AE} } \right)} \right) = \left( {\mathop \sum \limits_{\lambda = 300}^{{\lambda_{AE} }} \left( {1 - \frac{\lambda }{{\lambda_{AE} }}} \right) \cdot \varphi \left( \lambda \right)} \right) \cdot \alpha \cdot A \cdot I_{SW} $$

where *E*_*g*_ is bandgap energy, *E*_*thermalization*_ is thermalization energy, *E*_*p*_(*λ*_*AE*_) is the energy of absorption edge wavelength. Electrons at conduction band level then release their energy through non-thermal and thermal mechanisms. The thermal loss by stokes shift and quenching (*E*_*Stokes shift*+*quenching*_) loss can be calculated by subtracting PL energy (*E*_*PL*_) from band gap energy (*E*_*g*_):

13 $$ E_{Stokes shift + quenching} = E_{g} - E_{PL} = \left( {E_{p} \left( {\lambda_{AE} } \right) - E_{p} \left( {\lambda_{PL} } \right) \times QY} \right) \times \mathop \sum \limits_{\lambda = 300}^{{\lambda_{AE} }} NP_{absorbed} \left( \lambda \right) $$

Assuming a photoluminescent material with unity *QY*, the following equation for Stokes shift thermal loss can be derived:

14 $$ \begin{aligned} if\;QY & = 1 \Rightarrow E_{quenching} = 0 \Rightarrow E_{Stokes shift} \\ & = \left( {E_{p} \left( {\lambda_{AE} } \right) - E_{p} \left( {\lambda_{PL} } \right)} \right) \times \mathop \sum \limits_{\lambda = 300}^{{\lambda_{AE} }} NP_{absorbed} \left( \lambda \right) \\ & = \frac{SS}{{\uplambda_{AE} \cdot \left( {SS + \lambda_{AE} } \right)}} \cdot \left( {\mathop \sum \limits_{\uplambda = 300}^{{\uplambda_{AE} }} \uplambda \cdot \varphi \left( \uplambda \right)} \right) \cdot \alpha \cdot A \cdot I_{SW} \\ \end{aligned} $$

where *λ*_*PL*_ is the PL peak wavelength and *λ*_*AE*_ is the absorption edge wavelength. Also, the following equation for quenching heat loss can be obtained by subtracting Eq. (14) from Eq. (13):

15 $$ \begin{aligned} E_{quenching} & = E_{quenching + Stokes\;shift} - E_{Stokes\;shift} \\ & = E_{p} \left( {\lambda_{re} } \right) \times \left( {\mathop \sum \limits_{\lambda = 300}^{{\lambda_{AE} }} NP_{Absorbed} \left( \lambda \right) - \mathop \sum \limits_{\lambda = 300}^{{\lambda_{AE} }} QY\left( \lambda \right) \times NP_{absorbed} \left( \lambda \right)} \right) \\ & = \frac{1 - QY}{{SS + \lambda_{AE} }} \cdot \left( {\mathop \sum \limits_{\uplambda = 300}^{{\uplambda_{AE} }} \uplambda \cdot \varphi \left( \uplambda \right)} \right) \cdot \alpha \cdot A \cdot I_{SW} \\ \end{aligned} $$

## Results and discussion

We have employed our advanced model of non-radiative mechanisms to explore the impact of PL properties on transmission and thermal loss mechanisms. First, we estimated the impact of PL properties including *λ*_*AE*_, *QY*, and *SS* on thermal loss intensities. Then, the estimations were made to explore the most efficient photoluminescent material with minimal non-radiative recombination through both transmission and thermal mechanisms.

### Thermal loss

**Figure 2:**
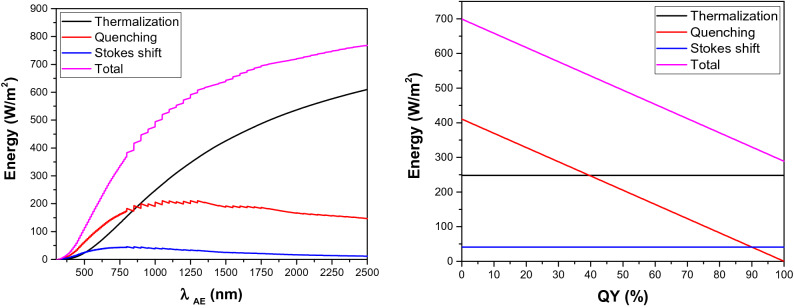
Left: Correlation between *λ*_*AE*_ and thermal loss mechanisms. *QY* and *SS* are kept constant at 50% and 100 nm, respectively. Right: Correlation between *QY* and thermal loss mechanisms. *λ*_*AE*_ and *SS* are kept constant at 1000 nm and 100 nm, respectively.

In this section, we estimated the impact of PL properties (i.e. *λ*_*AE*_, *QY*, and *SS*) on the three thermal loss mechanisms. Since we focus only on thermal loss mechanisms in this section, results can be used to enhance the photoluminescence cooling potential of integrated photoluminescence/NIR-reflective systems. The photoluminescence/NIR-reflective system is a two-layered heat-rejecting material composed of a nano-scale semiconductor photoluminescence material (QDs) as top coat to reject the UV and visible-range light and a NIR-reflective material as base coat^14^. The NIR-reflective layer in this system is used to minimize the transmission loss caused by highly absorptive conventional construction materials. First, we estimated the impact of *λ*_*AE*_ on the three thermal loss mechanisms. In this case, the *SS* and *QY* were kept constant at 100 nm and 50%, respectively. As shown in Fig. 2-left, Stokes shift loss has lower intensity than the quenching loss in the entire solar spectrum. Also, it has a lower contribution than that of thermalization loss at *λ*_*AE*_ longer than 507 nm. We have also calculated the contribution of each thermal loss mechanism for photoluminescent materials with different *λ*_*AE*_ and calculated the average contribution value in the entire solar spectrum, accordingly. As estimated, the contribution of Stokes shift, quenching, and thermalization is around 8.2%, 35%, and 56.8% of the total thermal loss. Also, the quenching and thermalization losses have almost the same intensity at *λ*_*AE*_ shorter than 843 nm. However, the thermalization loss is significantly higher than the quenching at longer wavelengths. Therefore, almost equal importance should be given to minimizing both quenching and thermalization for visible-emitting photoluminescent materials. As for NIR-emitting photoluminescent λmaterials, thermalization loss reduction through down conversion or tandem structure could be used as the most efficient method to enhance the photoluminescent cooling efficiency. Also, the impact of *QY* as a determining factor on quenching loss was estimated. In this case, *λ*_*AE*_ and *SS* was kept constant at 1000 nm and 100 nm, respectively. According to our estimations, for a given photoluminescent material with *λ*_*AE*_ at 1000 nm, quenching loss is lower than thermalization for *QY* efficiencies higher than 39% (See Fig. 2-right). Also, Stokes shift loss is lower than the quenching for *QY* efficiencies below 90%.

**Figure 3:**
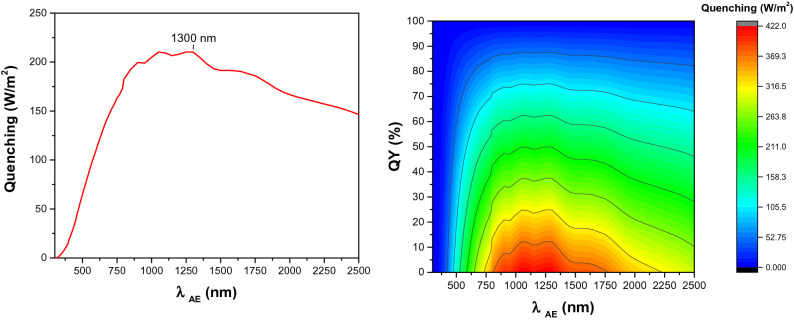
Left: The impact of *λ*_*AE*_ on quenching loss for a photoluminescent material with *QY* of 50%. Right: Correlation between quenching loss, *λ*_*AE*_ and *QY*. *SS* is kept constant at 100 nm for both cases.

In Fig. 3-left, the correlation between quenching loss and *λ*_*AE*_ for photoluminescent materials with *QY* of 50% is depicted. According to the estimations, the quenching loss reaches its maximum value at 1300 nm. The correlation between quenching loss, *λ*_*AE*_, and *QY* is also shown in Fig. 3-right. As per our results, the quenching loss shows its maximum value of 421.4 W/m^2^ for a photoluminescent material with *λ*_*AE*_ of 1300 nm and *QY* of close to zero.

**Figure 4:**
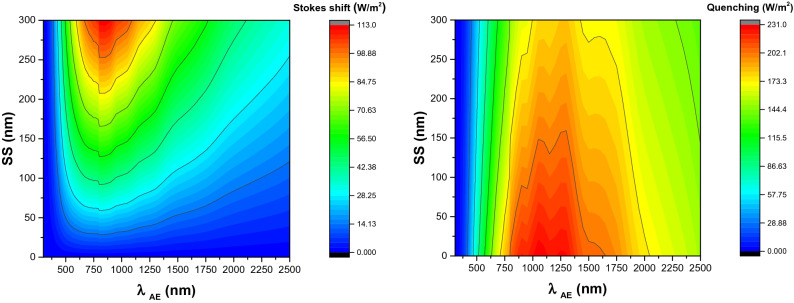
Left: Right: Correlation between Stokes shift thermal loss, *λ*_*AE*_, and *SS*. *QY* was kept constant at 50%. Right: Correlation between quenching loss, *λ*_*AE*_, and *SS*. *QY* was kept constant at 50%.

In parallel, we have employed our mathematical model to investigate the impact of *SS* and *λ*_*AE*_ on Stokes shift thermal loss. The estimations were made for photoluminescent materials with *SS* of up to 300 nm. According to our estimations, Stokes shift thermal loss has a maximum value of 112.6 W/m^2^ at *λ*_*AE*_ of around 850 nm and *SS* of 300 nm (See Fig. 4-left). Then, the impact of SS variation on quenching loss was computed. In contrast to Stokes shift loss, the quenching loss has a negative correlation with *SS* variation. The maximum quenching loss is estimated to be 230.6 W/m^2^ for a photoluminescent material with *SS* close to zero (See Fig. 4-right).

**Figure 5:**
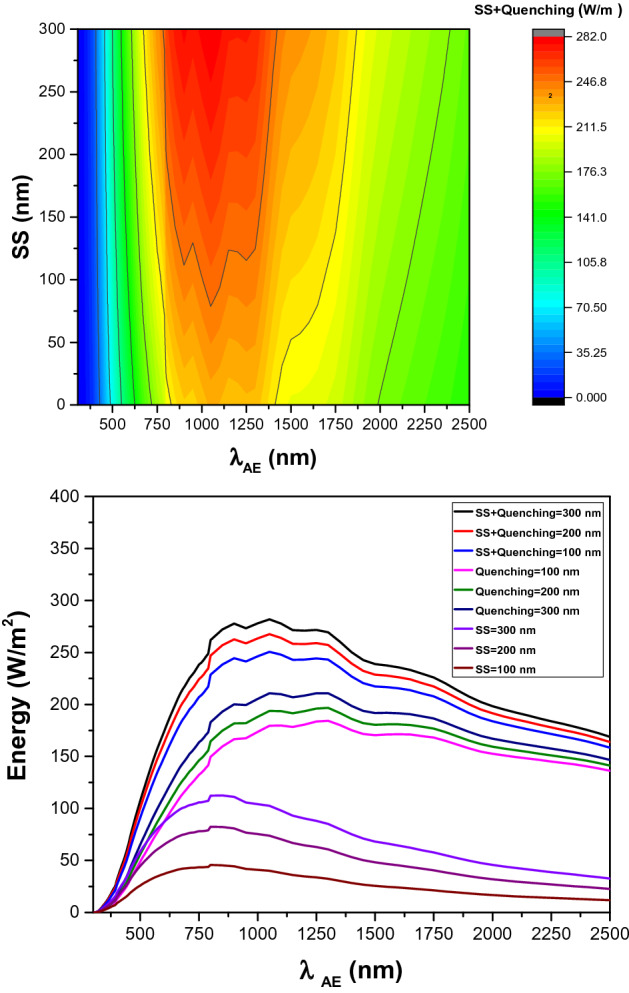
Top: Correlation between *SS*, *λ*_*AE*_, and total thermal loss by Stokes shift and quenching. Bottom: The Stokes shift loss, quenching loss, and total loss by Stokes shift and quenching for three different *SS* values of 100 nm, 200 nm, and 300 nm.

We have also computed the impact of *SS* variation on the overall loss by Stokes shift and quenching. As estimated, the *SS* variation has lower impact on the overall thermal loss by Stokes shift and quenching than the Stokes shift component. This can be explained by the negative correlation between SS variation and quenching loss component. Figure 5-top shows the correlation between *SS*, *λ*_*AE*_, and total thermal loss by Stokes shift and quenching. Figure 5-bottom illustrates the Stokes shift loss, quenching loss, and the total loss by Stokes shift and quenching for three different *SS* values of 100 nm, 200 nm, and 300 nm.

### Thermal and transmission loss

**Figure 6:**
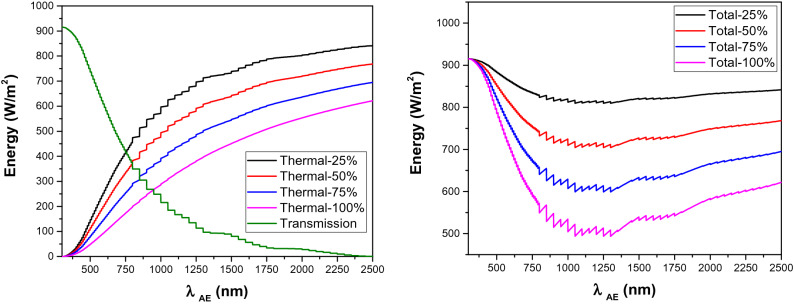
Left: Correlation between thermal and transmission losses with λ_AE_. Right: Correlation between total loss (i.e. thermal and transmission_)_ with λ_AE_.

Photoluminescence cooling as an additional cooling mechanism is capable of rejecting incidents lights with a wavelength shorter than the *λ*_*AE*_. All wavelengths longer than *λ*_*AE*_ are transmitted through the Photoluminescent layer, and therefore, thermal behaviour of the material depends on the optical properties of the substrate in this wavelength range. In this section, the correlation between thermal and transmission loss is investigated to explore the optimal PL properties for the maximum heat rejection potential in the whole solar spectrum. We have assumed to have a highly absorptive construction material as substrate. We first investigated the impact of *λ*_*AE*_ as the most important factor on thermal and transmission loss mechanisms to explore the optimal *λ*_*AE*_. The thermal loss estimations were made for four scenarios with four different *QY* values of 25%, 50%, 75%, and 100%. As indicated in Fig. 6-left, there is a sharp decrease in transmission loss within the visible range, which can be explained by the high portion of incoming light in this wavelength range. The transmission loss decreases at a much lower rate in the NIR range. On the other hand, thermal loss intensity increases with *λ*_*AE*_ mainly due to the thermalization loss component. The maximum transmission loss is predicted to be 640 W/m^2^ for a Photoluminescent material with the largest band gap (i.e. *λ*_*AE*_ of 300 nm). The maximum predicted thermal loss is 841, 768, 694, and 621 W/m^2^ for a Photoluminescence material with a very narrow bandgap at 2500 nm with *QY* of 25%, 50%, 75%, 100%, respectively. An equal share of non-radiative recombination through thermal and transmission is also foreseen for Photoluminescence materials with *λ*_*AE*_ of 750 nm and *QY* at 25%, *λ*_*AE*_ of 794 nm and *QY* at 50%, *λ*_*AE*_ of 850 nm and *QY* at 75%, and *λ*_*AE*_ of 949 nm and *QY* at 100%. The intersection points of thermal and transmission loss curves show the wavelengths above/below which up conversion/down conversion is the more effective measure to reduce the overall loss. The total loss by both thermal and transmission shows its minimum value at 1300 nm (See Fig. 6-Right). Overall, visible-emitting photoluminescent materials (i.e. *λ*_*AE*_ shorter than 700 nm) show higher overall non-radiative loss than their NIR-emitting counterparts.

## Conclusion

In this paper, an extended version of our PL cooling model for estimation of non-radiative processes of photoluminescent materials is developed. The main objective of this study is to investigate the impact of PL properties on non-radiative mechanisms to identify the most efficient methods to improve the PL cooling potential. The non-radiative recombination processes include thermal loss mechanisms for wavelengths equal or shorter than *λ*_*AE*_ and transmission loss at wavelengths longer than the *λ*_*AE*_. The three major thermal loss mechanisms include thermalization, quenching, and Stokes shift. The thermalization and transmission loss mechanisms are due to the mismatch between the absorbed photon energy and re-emitting photon energy, which could be rectified through development of down conversion and up conversion materials. Also, methods for improving the *QY* efficiency of photoluminescent nanoparticles (e.g. ligand exchange, core–shell structures, and embedment in polymer matrices) are the main strategy to minimize the thermal loss through quenching.

According to our results, transmission loss decreases with *λ*_*AE*_ at a much higher rate in the visible range than the NIR. On the contrary, thermal loss intensity increases with *λ*_*AE*_ in the whole solar spectrum. As predicted by our model, the thermal and transmission loss curves intersect each other at wavelengths between 750 and 949 nm for photoluminescent materials with a *QY* efficiency between 25 and 100%. The intersection point shows the wavelength where either down conversion or up conversion processes should be used to improve the PL cooling efficiency. Also, NIR-emitting photoluminescent materials have much higher sensitivity to *QY* variation than the visible-emitting counterparts. More specifically, photoluminescent materials with a *λ*_*AE*_ at 1300 nm show the maximum quenching loss reduction by *QY* improvement. Our model also predicts that Stokes shift loss has the lowest intensity compared to all other non-radiative mechanisms.
